# Supplementary figures and images for: Molecular Epidemiology Surveillance of SARS-CoV-2: Mutations and Genetic Diversity One Year after Emerging
Source: Pathogens. 2021 Feb 9;10(2):184. doi: 10.3390/pathogens10020184 (PMC7915391; doi:10.3390/pathogens10020184)

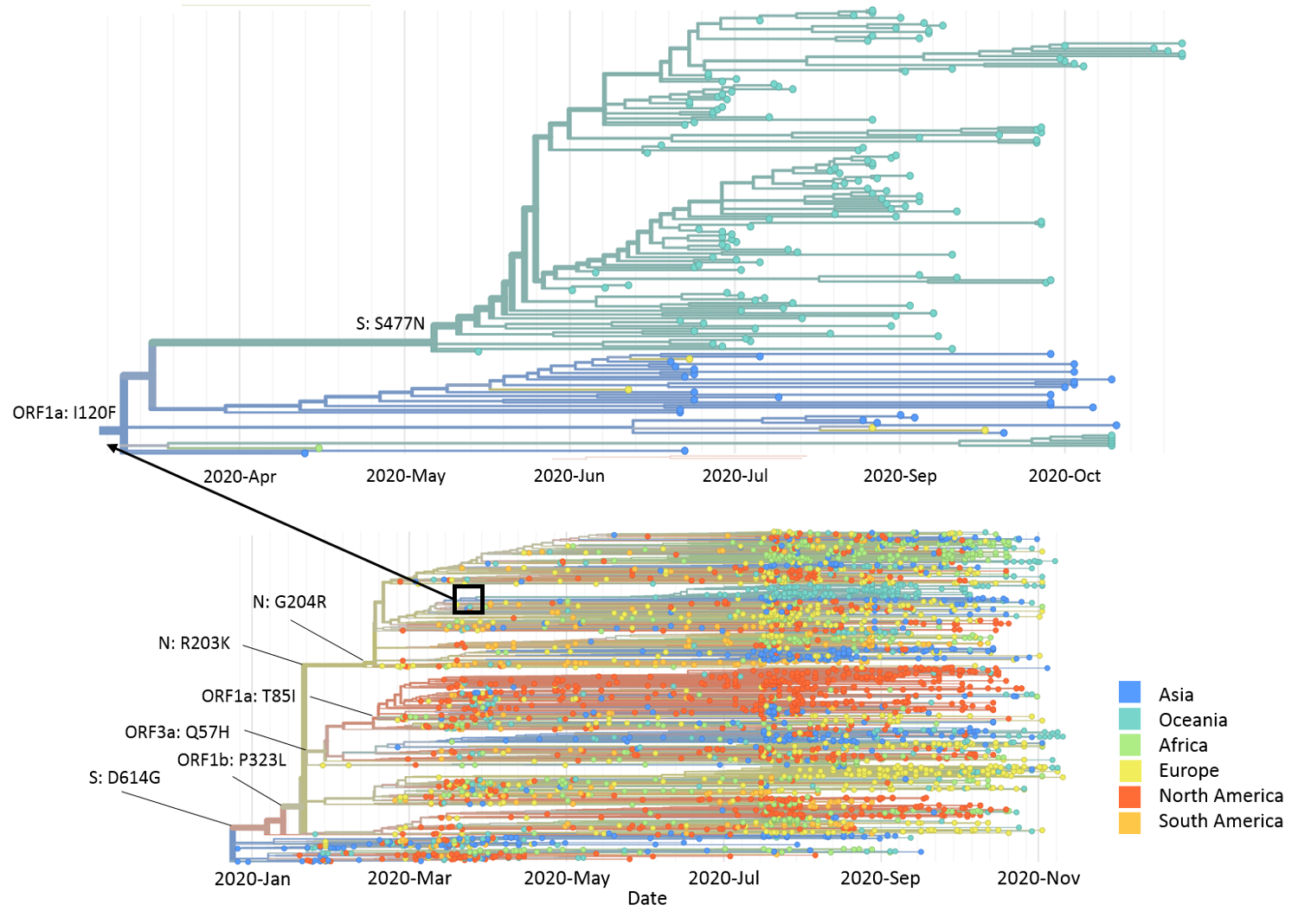

Supplement: Supplementary file 1 [file pathogens-10-00184-s001.zip › Figure S1.tif]
